# The K_Ca_2 Channel Inhibitor AP30663 Selectively Increases Atrial Refractoriness, Converts Vernakalant-Resistant Atrial Fibrillation and Prevents Its Reinduction in Conscious Pigs

**DOI:** 10.3389/fphar.2020.00159

**Published:** 2020-02-28

**Authors:** Jonas Goldin Diness, Jeppe Egedal Kirchhoff, Tobias Speerschneider, Lea Abildgaard, Nils Edvardsson, Ulrik S. Sørensen, Morten Grunnet, Bo Hjorth Bentzen

**Affiliations:** ^1^ Department of In Vivo Pharmacology, Acesion Pharma, Copenhagen, Denmark; ^2^ Department of Biomedical Sciences, Faculty of Health and Medical Sciences, University of Copenhagen, Copenhagen, Denmark; ^3^ Department of Molecular and Clinical Medicine/Cardiology, Institute of Medicine, Sahlgrenska Academy, University of Gothenburg, Gothenburg, Sweden

**Keywords:** atrial fibrillation, ion channels, antiarrhythmic drugs, SK channels, K_Ca_2

## Abstract

**Aims:**

To describe the effects of the K_Ca_2 channel inhibitor AP30663 in pigs regarding tolerability, cardiac electrophysiology, pharmacokinetics, atrial functional selectivity, effectiveness in cardioversion of tachy-pacing induced vernakalant-resistant atrial fibrillation (AF), and prevention of reinduction of AF.

**Methods and Results:**

Six healthy pigs with implanted pacemakers and equipped with a Holter monitor were used to compare the effects of increasing doses (0, 5, 10, 15, 20, and 25 mg/kg) of AP30663 on the right atrial effective refractory period (AERP) and on various ECG parameters, including the QT interval. Ten pigs with implanted neurostimulators were long-term atrially tachypaced (A-TP) until sustained vernakalant-resistant AF was present. 20 mg/kg AP30663 was tested to discover if it could successfully convert vernakalant-resistant AF to sinus rhythm (SR) and protect against reinduction of AF. Seven anesthetized pigs were used for pharmacokinetic experiments. Two pigs received an infusion of 20 mg/kg AP30663 over 60 min while five pigs received 5 mg/kg AP30663 over 30 min. Blood samples were collected before, during, and after infusion on AP30663. AP30663 was well-tolerated and prominently increased the AERP in pigs with little effect on ventricular repolarization. Furthermore, it converted A-TP induced AF that had become unresponsive to vernakalant, and it prevented reinduction of AF in pigs. Both a >30 ms increase of the AERP and conversion of AF occurred in different pigs at a free plasma concentration level of around 1.0–1.4 µM of AP30663, which was achieved at a dose level of 5 mg/kg.

**Conclusion:**

AP30663 has shown properties in animals that would be of clinical interest in man.

## Introduction

An effective, safe, and tolerable pharmacological treatment for atrial fibrillation (AF) remains an unmet need.

The K_Ca_2 channels, also known as small conductance Ca^2^^+^-activated K^+^, or SK channels have emerged as a promising new target for AF treatment. Inhibition of K_Ca_2 channels with different compounds converted AF and/or protected against its induction in models of AF in isolated perfused hearts from rat, guinea pig, and rabbit as well as in *in vivo* models of AF in rat, dog, pig, goat, and horse (Diness et al., 2010; Diness et al., 2011; Skibsbye et al., 2011; Qi et al., 2013; Haugaard et al., 2015; Diness et al., 2017; Gatta et al., 2019). The small molecule AP30663 selectively inhibits K_Ca_2 channels thereby diminishing the current mediated by them, I_KCa_ (Bentzen et al., 2020). Other small-molecule K_Ca_2 channel inhibitors give rise to adverse central nervous effects such as vomiting and tremors (Diness et al., 2017; Simó-Vicens et al., 2017), but this does not seem to be the case with AP30663.

The aim of this manuscript is to describe the effects of AP30663 in pigs with regard to tolerability, cardiac electrophysiology, pharmacokinetics, atrial functional selectivity, effectiveness in cardioversion of tachy-pacing induced vernakalant-resistant AF, and prevention of reinduction of AF.

## Materials and Methods

All animal studies were performed under a license from the Danish Ministry of Environment and Food (license No. 2014-15-0201-00390) and in accordance with the Danish guidelines for animal experiments according to the European Commission Directive 86/609/EEC.

**Figure 1:**
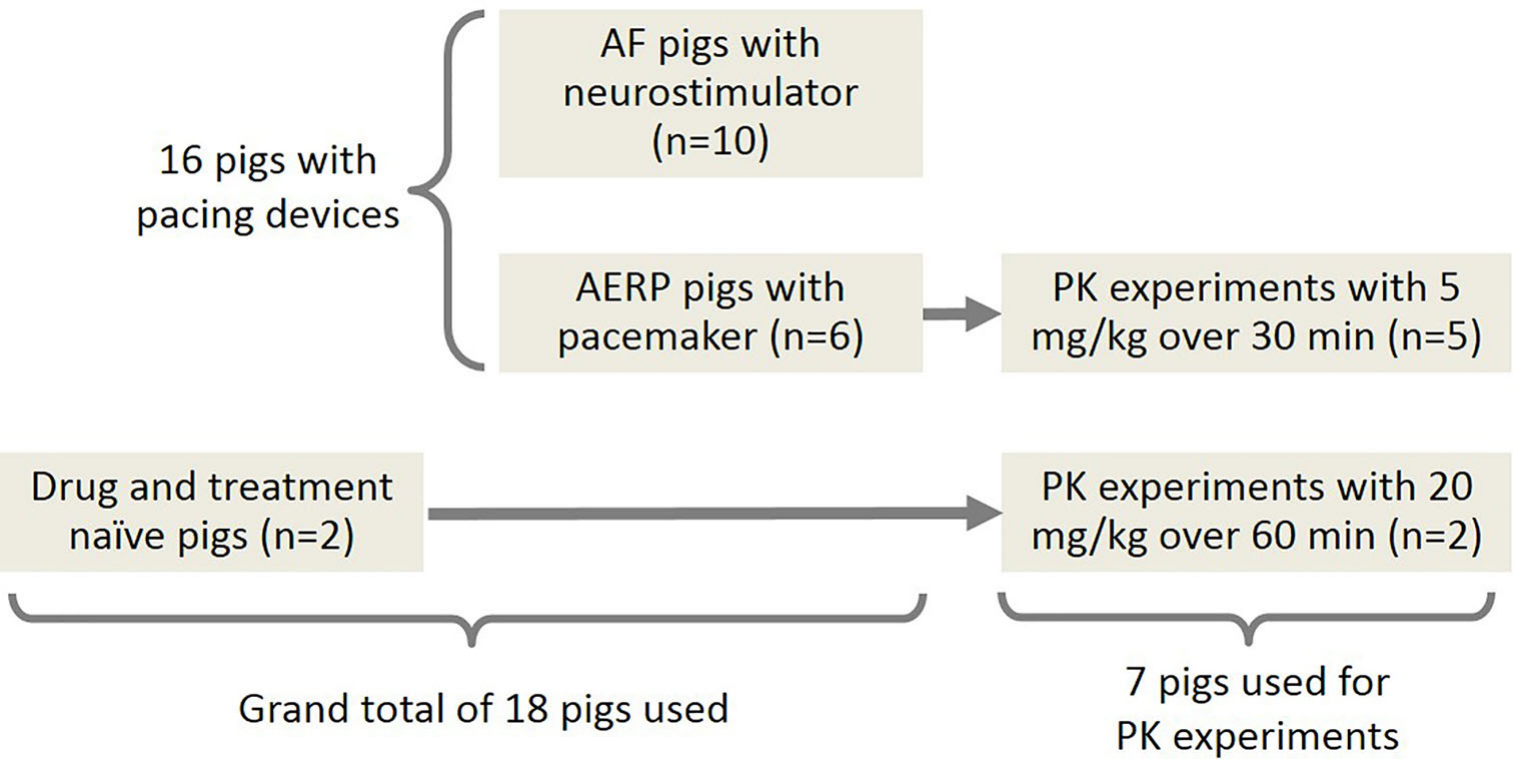
Overview of the experiments and the participating pigs. A-TP pigs: pigs subjected to AF induction by atrial tachy-pacing; AERP pigs: pigs for determination of the AERP.

An overview of the experiments and the participating pigs is presented in **Figure 1**. Detailed tables of the equipment, materials, and software used in this study can be found in the **Supplementary Materials**.

### Implantation of Leads and Pacemakers/Neurostimulators in Pigs

Sixteen Danish female landrace pigs had cardiac pacing devices implanted at an age of 11 weeks (25–35 kg). The pigs underwent the following procedure: After premedication with zoletil pig mixture, the pig was given an infusion of propofol and fentanyl (15 mg/kg/h and 50 µg/kg/h respectively) and intubated and ventilated with a tidal volume of 10 ml/kg and a respiration frequency of 12–14/min. During surgery PaCO_2_, blood pressure, and electrocardiography (ECG) were monitored, and the pig was given 6 ml/kg/h isotonic salt solution. Under aseptic conditions and fluoroscopic guidance, one or two bipolar pacing-electrode leads were inserted into the right-atrium and connected to a pacing device implanted in the neck. The six pigs that were used for atrial effective refractory period (AERP) and QT recordings had two electrodes implanted that were connected to a pacemaker (Biotronik, Etrinsa DR-T). The ten pigs used for induction and cardioversion of AF (2.3) had one electrode implanted that was connected to a neurostimulator (Medtronic, Synergy Versitrel or Itrel3). An ear vein catheter was also placed in all the pigs in these experiments.

### AERP and QT Recordings From Conscious Pigs

In order to test the minimal efficacious dose of the K_Ca_2 channel inhibitor AP30663, a healthy conscious pig model was used in which an implanted pacemaker with diagnostic functions allowed measurements of AERP, a surrogate efficacy parameter for conversion of AF. Furthermore, the pigs were equipped with a Holter monitor (Televet 100) that allowed online monitoring and recording of the ECG.

With at least 48 h intervals each pig received an infusion over 30 min of vehicle or 5, 10, 15, 20, or 25 mg/kg AP30663. The dosing always took place post-meal and started between 9 am and 2 pm.

The AERP was measured every 5^th^ minute (± 2 min) throughout the experiment, from 15 min prior to drug infusion to 30 min after the end of infusion, by applying trains of 10 pacing stimuli with a fixed interval of 330 ms (S1) followed by an extra stimulus (S2) applied with 10 ms increments starting from 80 ms. Each train of S1 and S2 stimuli was separated with 1 s. The AERP was defined as the longest S1–S2 interval failing to elicit a response. The lowest dose to produce an AERP prolongation of ≥30 ms was defined as the minimal efficacious dose.

From the ECG, the QT interval, a surrogate safety parameter, was analyzed at (in minutes ±2 min from the start of infusion): −15, −10, -5, −2, 15, 30, 45, 60. The unpaced QT interval was analyzed by taking an average of a suitable ECG lead over 50 beats at intrinsic heart rate (HR). A study specific QT correction formula was generated by plotting baseline pairs of QT intervals and HRs and fitting them with linear regression, enabling calculation of an “expected QT” based on the heart rate (Expected QT = y-intercept – slope * HR). The QT interval corrected for HR (QTc) was defined as [200 ms + (actual QT−expected QT)]. All ECGs were analyzed semiautomatically with the ECG analysis module in LabChart Pro v. 7.3.8 with manual verification.

At least 48 h after the last dosing was completed a terminal experiment was conducted in anesthetized pigs. During the terminal experiment, each pig was treated with the minimal efficacious dose of AP30663, and the pharmacokinetic properties of AP30663 were examined by taking blood samples during and after the infusion.

### Induction of AF, Cardioversion of AF and Reinduction of AF in Pigs

A previously described *in vivo* pig model was used, in which long-lasting atrial tachy-pacing was performed in order to develop sustained AF that was resistant to pharmacological cardioversion with a clinically relevant dose of vernakalant (Diness et al., 2017). In this model of sustained vernakalant-resistant AF, the K_Ca_2 channel inhibitor AP30663 was infused with a rate of 20 mg/kg/h over 60 min, to discover if it could successfully convert AF to SR and protect against reinduction of AF in conscious pigs.

**Figure 2:**
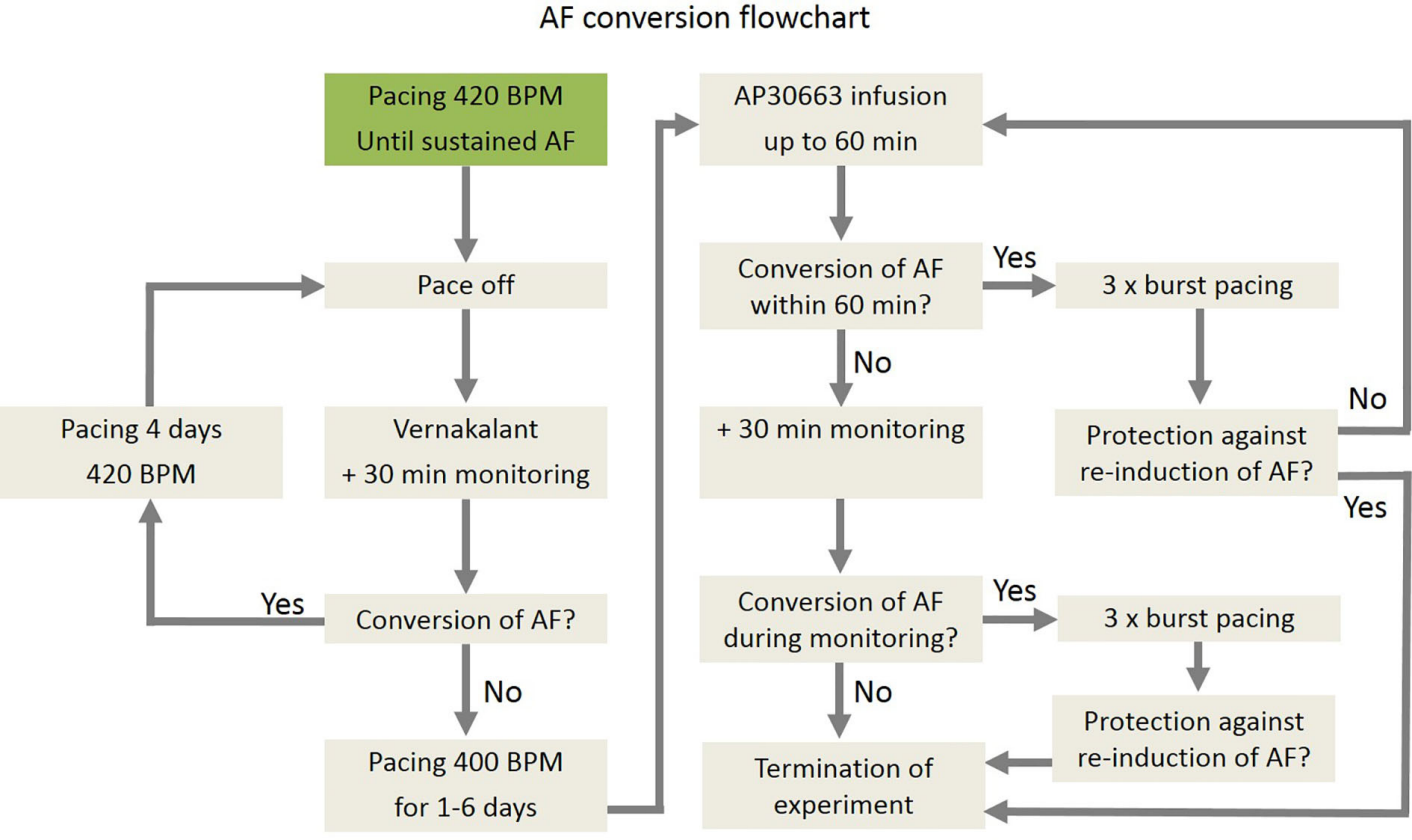
Flow chart of induction of AF, cardioversion of AF, and reinduction of AF in pigs.

A total of ten pigs were used for induction and cardioversion of AF. An overview of the study plan is described in **Figure 2**. A more detailed description can be found in the **Supplementary Materials**.

### Pig Pharmacokinetics

#### Preparation of Closed Chest Pharmacokinetics Pigs

A total of seven Danish landrace pigs (gilts) were used for the PK experiments. The pigs underwent the following procedure: After premedication with zoletil pig mixture, the pig was given an infusion of propofol and fentanyl (15 mg/kg/h and 50 µg/kg/h respectively) and intubated and ventilated with a tidal volume of 10 ml/kg and a respiration frequency of 12–14/min. During surgery, PaCO_2_, blood pressure, and ECG were monitored, and the pig was given 6 ml/kg/h isotonic salt solution.

Two 11 weeks old (27–33 kg), drug-naïve pigs received an infusion of 20 mg/kg AP30663 over 60 min. Five of the six pigs that had previously received AP30663 for AERP and QT recordings received 5 mg/kg AP30663 over 30 min (**Figure 1**). The drug infusions were given *via* the right external jugular vein, starting from t = 0.

#### Plasma Sampling

During the experiment blood samples were taken from a femoral vein for PK-analyses. For the two pigs that received 20 mg/kg AP30663 over 60 min blood was sampled at the following times (in minutes from the beginning of infusion): −10, 5, 10, 15, 20, 30, 40, 50, 60, 65, 70, 80, 90, 100, 120, 140, 160, and 180. For the pigs that received 5 mg/kg over 30 min, blood was sampled at the following times (in minutes from the beginning of infusion): −10, 5, 10, 15, 20, 25, 29, 35, 40, 50, and 60.

For each sample, the first 10 ml of blood was discarded and 10 ml was stored in a heparin coated blood collection tube, gently shaken, and centrifuged at 2,000 G for 4 min at 4°C. The plasma separated from the blood were stored in 2 ml aliquots in Eppendorff tubes and frozen (−18°C). For analyses, the plasma samples were sent to Syngene (Bangalore, India) stored on dry ice with a temperature tracker and stayed below −20°C for the entire time until analysis.

### Drugs and Solutions

Unless otherwise mentioned, all the chemicals used were of analytic grade and were obtained from Sigma-Aldrich. See **Supplementary Tables 2** **–** **4** in the **Supplementary Materials** for details.

#### Preparation of Test Drug

Vehicle 1 consisted of 50% polyethylene glycol 400 (PEG) and 50% isotonic sterile saline water for injection. 10 mg/ml AP30663 was dissolved in PEG, and saline was added q.s. to yield a final concentration of 5 mg/ml. Vehicle 1 was used for the two PK pigs that received 20 mg/kg AP30663 and for AF cardioversion.

Vehicle 2 was an aqueous solution with a pH of 4.5 with a final concentration of 10 mg/ml AP30663. Vehicle 2 was used for the AERP and QT recordings from conscious pigs and for the PK pigs that received 5 mg/kg AP30663.

All solutions of the test compound for *in vivo* injection were sterilized by a final filtration at 0.22 µm (Nylon filter).

### Statistical Analysis

Statistical analysis and drawings were performed using GraphPad Prism software.

Continuous data are summarized using the mean ± SEM. One-way repeated measures ANOVA assuming sphericity with Sidak's multiple comparisons *post hoc* test was used to compare ΔAERP, ΔQTc, and ΔHR from start to end of infusion of AP30663 to the same values from start to end of infusion of vehicle. In order to test for a linear dose–response relationship, the values of ΔAERP, ΔQTc, and ΔHR from start to end of infusion of AP30663 were analyzed as a function of dose with simple linear regression. Departure from linearity was tested with a replicates test. P values are given with three decimals.

#### Fitting of PK Curves

The plasma concentrations during infusion for 20 mg/kg AP30663 over 60 min and 5 mg/kg AP30663 over 30 min were fitted as a one phase association with this formula:

C=C0+(Plateau−C0)*[1−exp(−K*T)]

Where C is the plasma concentration, T is time, and K is the elimination rate constant with two constraints: that the initial concentration (C0) must be zero and K must be shared between the two data sets. The plasma concentrations after the end of infusion were fitted as a two-phase decay with this formula:

C=C0*PercentFast*0.01*exp(−Kd*T)+C0*(100−PercentFast)*0.01*exp(−Ke*T)

Where T is time, C0 is the concentration at T = 0, Kd is the distribution rate constant, Ke is the elimination rate constant, and PercentFast is the fraction of the span (from C0 to zero) accounted for by the faster of the two components. There were two constraints: the Kd- and the Ke-values must be shared between the two data sets.

## Results

### AERP Recordings From Healthy Conscious Pigs

A total of six female healthy landrace pigs were dosed with the following doses of AP30663 given as a constant rate infusion over 30 min (in mg/kg): 0, 5, 10, 15, 20, and 25. The different doses were administered with at least 48 h intervals. All doses in all pigs were well tolerated, and the associated average AERP measurements before, during and after infusion can be seen in **Figure 4A**. In **Table 1** the average differences in AERP, QTc, and HR from the start to the end of infusion have been listed. The lowest tested dose of AP30663 (5 mg/kg) was also the lowest dose to produce an AERP prolongation of ≥30 ms and was thus defined as the minimal efficacious dose. The test Linear regression of the average differences in AERP from the start to the end of infusion as a function of dose indicates that there was a linear and relatively steep dose dependent effect of AP30663 on AERP (**Figure 5A**).

**Table 1:** Average values of ΔAERP, ΔQTc, and ΔHR from the start to the end of infusion of AP30663.

AP30663 dose (mg/kg)	ΔAERP				ΔHR				ΔQTc (ms)	P-value
	Ms	p-value	%	p-value	bpm	p-value	%	p-value		
0	10 ± 3	NA	11 ± 3	NA	−3 ± 4	NA	−2 ± 3	NA	−0.4 ± 1.7	NA
5	35 ± 6	0.047	33 ± 5	0.089	−13 ± 3	0.300	−8 ± 2	0.351	7.3 ± 1.7	0.277
10	43 ± 8	0.005	42 ± 9	0.008	−23 ± 5	0.003	−15 ± 3	0.006	3.5 ± 3.1	0.864
15	57 ± 8	<0.001	50 ± 7	<0.001	−27 ± 5	<0.001	−19 ± 4	<0.001	6.3 ± 3.4	0.418
20	65 ± 6	<0.001	56 ± 5	<0.001	−22 ± 4	0.005	−15 ± 3	0.006	7.9 ± 3.0	0.216
25	83 ± 7	<0.001	71 ± 5	<0.001	−14 ± 5	0.187	−11 ± 4	0.120	10.5 ± 3.8	0.055

Paired comparison of values after infusion of AP30663 to values after infusion of vehicle, n = 6. AERP, Atrial effective refractory period; QTc, Heart rate corrected QT interval, study specific formula; HR, Heart rate.

### Effects on HR and QT Intervals in Conscious Pigs

**Figure 3:**
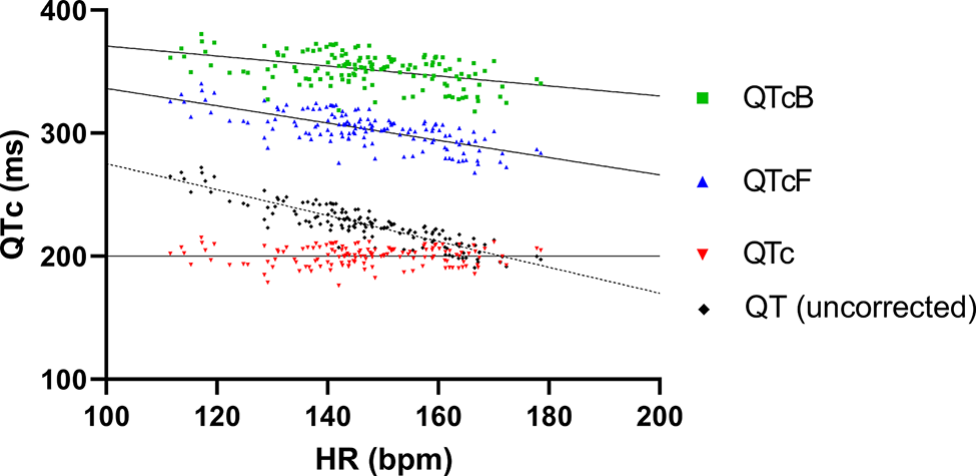
QT and different QT correction formulas applied on baseline recordings plotted as functions of heart rate. Linear regression of both Bazett's and Fridericia's formulas (QTcB and QTcF, respectively) revealed slopes that significantly deviated from zero (−0.41 ± 0.07 and −0.70 ± 0.06, respectively). In comparison the slope of linear regression of the study-specific formula was <0.0001 ± 0.04 which was not significantly different from zero.

The ECG recordings from the six pigs that were dosed as described in 3.1 were analyzed at intrinsic HR. To obtain best possible correction of QT intervals to HR of the animals included in the study, all unpaced baseline pairs of QT intervals and HRs were plotted and fitted with linear regression (**Figure 3**), resulting in the following function:

Expected QT=380.5 ms−1.053*HR.

Thus, a study specific formula for the QT interval corrected for HR (QTc) could be made:

QTc=200 ms+(actual QT−expected QT).

The QT intervals were also corrected for HR with Bazett's and Fridericia's formulas, but when plotting QTcB and QTcF against HR linear regression revealed a significantly nonzero slope, indicating that these formulas provided inadequate correction (**Figure 3**). Thus, only the study specific formula for HR correction of the QT interval was used for analysis.

**Figure 4:**
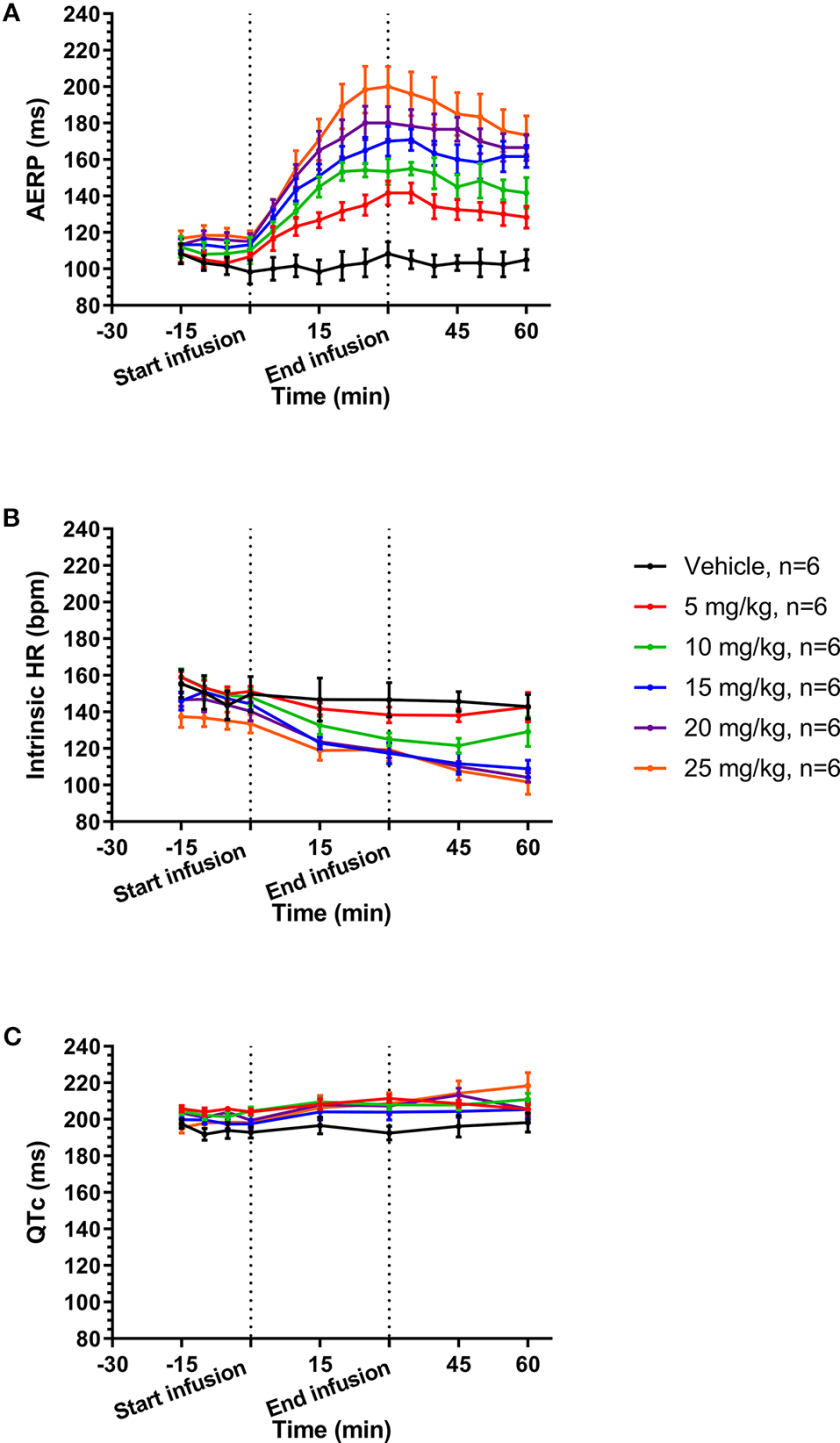
Average values before, during, and after infusion of increasing concentrations of AP30663 of atrial effective refractory period (AERP) **(A)**, intrinsic heart rates **(B)**, and QTc **(C)**.

The average values of QTc and intrinsic HRs before, during, and after infusion can be seen in **Figure 4B** and **Figure 4C**. In **Table 1** the average ΔQTc and ΔHR from the start to the end of infusion have been listed. When looking at individual doses, QTc increased by an average of 10.5 ± 3.8 ms at the highest dose, and this change could, but need not, be the result of the drug and not just biological variation (p = 0.055). At the other individual dose levels the QTc was only increased to a minor extent and could be attributed to biological variation.

**Figure 5:**
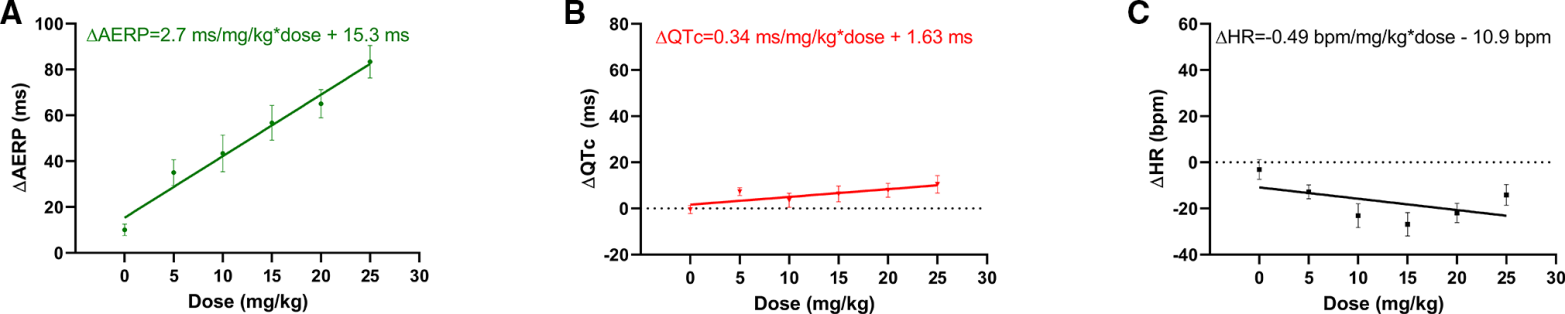
Simple linear regression of **(A)** ΔAERP, **(B)** ΔQTc, **(C)** ΔHR from start to end of infusion of AP30663 as a function of dose with simple linear regression. Departure from linearity was tested with a replicate test. All slopes were significantly different from zero (p < 0.001, p = 0.018, and p = 0.49 for ΔAERP, ΔQTc, and ΔHR, respectively). No evidence was found of nonlinearity for ΔAERP and ΔQT as functions of dose (p = 0.721, and p = 0.604, respectively), but ΔHR as a function of dose deviated significantly from linearity (p = 0.019). AERP, Atrial effective refractory period; QTc, QT interval corrected for heart rate, HR, Heart rate.

Linear regression of the average differences in QTc from the start to the end of infusion as a function of dose, however, indicates that there was a linear, dose dependent effect of AP30663 on QTc (**Figure 5B**).

The intrinsic HR decreased by 13 to 27 beats/min (8 to 27%) with no apparent dose and/or concentration relationship (**Table 1**).

Linear regression of the average differences in HR from the start to the end of infusion as a function of dose indicates that there was a linear, dose dependent effect of AP30663 on AERP (**Figure 5C**).

### Pig Pharmacokinetics

**Figure 6:**
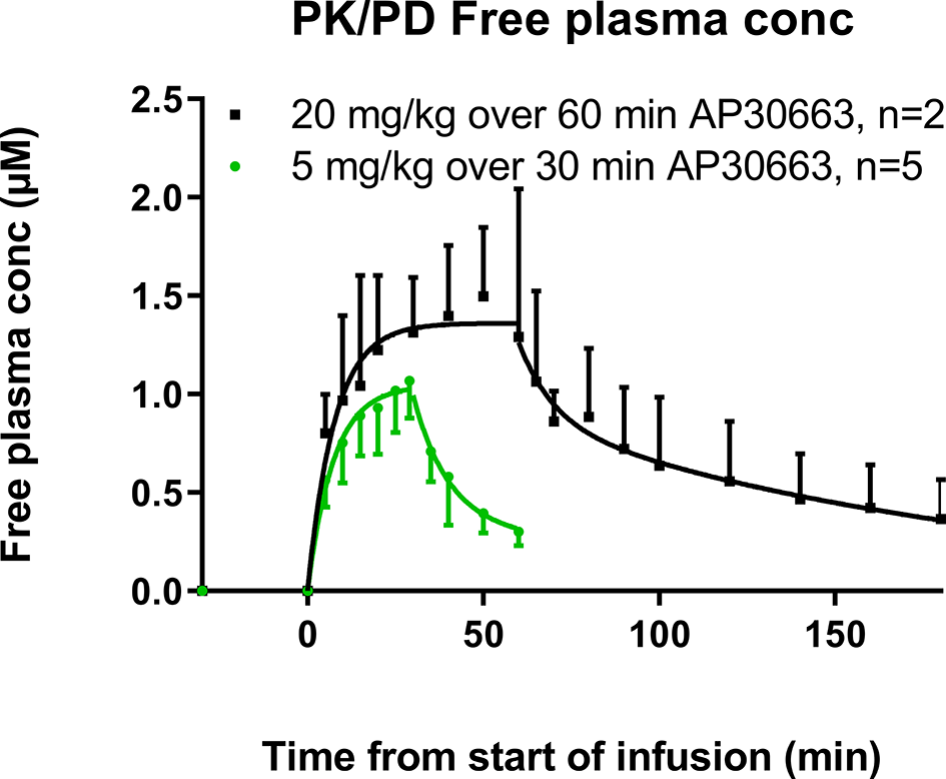
Free plasma concentrations of AP30663 at different time points before, during, and after infusion. The plasma concentrations during infusion were fitted as a one phase association with this formula: C = C0 + (Plateau−C0) * [1-exp(−K * T)], where C is the plasma concentration, T is time, and K is the elimination rate constant with two constraints: that the initial concentration (Y0) must be zero and K must be shared between the two data sets. The plasma concentrations after the end of infusion were fitted as a two-phase decay with this formula: C = C0 * PercentFast * 0.01 * exp(−Kd * T) + C0 * (100−PercentFast) * 0.01 * exp(−Ke * T), where T is time, C0 is the concentration at T = 0, Kd is the distribution rate constant, Ke is the elimination rate constant, and PercentFast is the fraction of the span (from C0 to zero) accounted for by the faster of the two components. There were two constraints: the Kd- and the Ke-values must be shared between the two data sets.

Two drug-naïve pigs received an infusion of 20 mg/kg AP30663 over 60 min. Five of the six pigs that had previously received AP30663 for AERP and QT recordings received 5 mg/kg AP30663 over 30 min (**Figure 1**). In **Figure 6** the calculated free plasma concentration is plotted at different time points before, during, and after infusion.

As would be expected with an i.v. infusion, the maximal plasma concentration (Cmax) was observed at the end of infusion for both groups of pigs. The Cmax were 3360 ± 113 ng/ml and 4532 ± 844 ng/ml for the pigs that received 5 mg/kg and 20 mg/kg AP30663, respectively. The fraction of AP30663 that binds to the plasma proteins in landrace pigs was 0.864. Combined with a molar weight of 401.39 g/mol the free Cmax values were 1.14 ± 0.04 µM and 1.54 ± 0.29 µM for the two dose groups.

The plasma concentrations during infusion were described well by first-order kinetics with a half-time of 5.2 min, whereas the plasma concentrations after infusion followed the Michaelis–Menten kinetics with a distribution half-life of 6.8 min and an elimination half-life of 93.7 min. Thus, it seems that there is a fast distribution to other tissues and an elimination half-life of approximately 90 min. The plasma concentration during infusion must be controlled primarily by the fast distribution to body tissues and only to a limited extent by the elimination half-life.

### AF Conversion

Six out of ten pigs with vernakalant-resistant AF cardioverted during infusion of 20 mg/kg AP30663 over 60 min. Four out of these six pigs were protected against reinduction of AF by burst pacing. The average time to cardioversion was 29 ± 18 min corresponding to calculated free plasma concentrations of 1.0–1.4 µM AP30663. In all the conscious pigs AP30663 was well tolerated with no adverse events.

## Discussion

The K_Ca_2 inhibitor AP30663 was well-tolerated and prominently increased the AERP dose- and plasma concentration-dependence in pigs with little effect on ventricular repolarization. Furthermore, it converted A-TP induced AF that had become unresponsive to clinically relevant doses of vernakalant (4 mg/kg infused over 10 min), and it prevented reinduction of AF in pigs. Both a >30 ms increase of the AERP and conversion of AF occurred, in different pigs, at a free plasma concentration level of around 1.0–1.4 µM of AP30663, which was achieved already at a dose level of 5 mg/kg.

### Pronounced Increase of AERP at High Paced HRs in Pigs

The average intrinsic HR of pigs was 147 ± 14 bpm at baseline and 181 bpm was used for overdrive pacing. The AERP continued to increase when increasing doses were given to the same pigs on different days. A ≥30 ms increase of the AERP was used as a surrogate efficacy parameter and was reached already at a dose of 5 mg/kg where the AERP was 142 ± 7 ms *vs.* baseline 107 ± 2 ms. At 20 and 25 mg/kg the AERP had increased by 65 ± 6 ms and 83 ± 7 ms or 57 and 71%. The fact that significant increases of the AERP were obtained at a paced HR as high as 181 bpm supports that it could be of clinical significance.

We assumed that a ≥30 ms increase of the AERP would be sufficient for the conversion of AF since this was previously achieved by a clinically relevant dose of vernakalant in anesthetized male pigs (Bechard et al., 2011). Specifically, a vernakalant loading dose of 7.2 mg/kg infused over 60 min followed by a maintenance dose of 3.6 mg/kg infused over 45 min producing a steady plasma concentration of 4127 ± 194 ng/ml (11.8 ± 0.6 μM) caused an increase in AERP to 215 ± 5 ms from a baseline of 181 ± 7 ms corresponding to 34 ± 8 ms or 19% (Bechard et al., 2011). In patients with AF lasting 3 h to 7 days, the same plasma concentration resulted in 51.7% of the patients converting to sinus rhythm compared with 4.0% in the placebo group (Roy et al., 2008; Mao et al., 2009).

The current results support that an AERP increase of ≥30 ms in pigs is a meaningful measure for predicting cardioversion efficacy since infusion of 5 mg/kg AP30663 over 30 min produced a free plasma concentration of 1.14 ± 0.04 µM in our PK pigs which is within the concentration range of 1.0–1.4 µM where conversion to sinus rhythm was observed in the A-TP induced AF pigs. Since cardioversion takes place at a concentration that corresponds to the *in vitro* K_Ca_2 IC_50_ value (Bentzen et al., 2020), *i.e.* when ~50% of the I_KCa_ is blocked, it could be speculated that there is room for better effects at higher plasma concentrations of AP30663. The possibility of obtaining a conversion to sinus rhythm by I_KCa_ inhibition with AP30663 might also remain even in a setting of reduced K_Ca_2 expression since it would still be possible to inhibit I_KCa_ enough to obtain conversion.

### Decrease in HR — Little Effect on the QT Interval

The HR decreased significantly (**Table 1**) by up to 27 bpm (19%) without an apparent dose or concentration dependent pattern. It also did not correlate with dose-dependent increase of the AERP.

The ECG lead placement in the pigs provided ECGs of high quality that enabled reliable measurement of the QT interval with semiautomatic analysis with the possibility of manual overriding.

A study specific formula for correcting the QT interval for HR was made since both Bazett's and Fridericia's formulas provided insufficient correction for the influence of HR on the QT interval. Thus, at low HRs these formulas would be expected to report too large QT intervals and too small QT intervals at fast HRs.

The study specific QTc increased to a maximum ΔQTc of 10.5 ms at 25 mg/kg, while there was a decrease in the HR by 14 bpm compared to control. At this dose level the increase in QTc could probably be attributed to AP30663 and not to biological variation even though the p-value was 0.055. However, the linear regression revealed that there was a linear and dose dependent effect on QTc, however small.

The decrease in HR was less likely to be attributed to AP30663 (p = 0.187 for the highest dose). It is not possible to determine to what extent, if any, AP30663 might have been involved. Fitting ΔHR as a function of AP30663 dose with linear regression was not conclusive since it deviated significantly from linearity (p = 0.019).

An increase of the QT interval would not be improbable, considering that AP30663 was associated with a K_V_11.1 IC_50_ of 15 and 4 µM in two different analyses (Bentzen et al., 2020).

### Conversion of AF in the Vernakalant-Resistant Pig Model and Reinduction of AF

Persistent AF was induced by A-TP after a mean of 17 days. Vernakalant 4 mg/kg was given, and if conversion to sinus rhythm was obtained, pacing was continued, and vernakalant was repeated 3–7 days later. When vernakalant had become ineffective and had been washed out, AP30663 was given and succeeded in conversion to sinus rhythm in six out of 10 pigs.

It is well-known that in patients the efficacy of vernakalant declines with increasing AF duration. In the ACT I trial vernakalant converted 62% of the patients with AF lasting for 3 to 48 h, 24% of the patients with AF lasting 3 to 7 days and 8% of the patients with AF lasting 8 to 45 days, the latter being no better than the placebo (Roy et al., 2008).

We assume that our current A-TP pig model of AF creates a substrate that continues to develop until vernakalant no longer works. The fact that AP30663 was able to convert AF at this stage implies that AP30633 may be of clinical importance also when electrical and structural remodeling has taken place, which is likely to be the case in most patients with AF. This is further supported by the failure of attempts to reinduce AF.

### Functional Atrial Selectivity

Effective atrial selective antiarrhythmic therapy with a lower risk of ventricular proarrhythmia has been a long sought-for goal. Inhibition of the atrial-specific ultrarapid delayed rectifier current (I_Kur_) or the acetylcholine-activated inward-rectifier potassium current and (I_K,ACh_) was among the first of these approaches, but most compounds targeting these currents have been discontinued after unsuccessful initial clinical trials (Heijman and Dobrev, 2017; Shunmugam et al., 2018; Camm et al., 2019).

As the first K_Ca_2 inhibitor to enter clinical trials, AP30663 was selected to be the lead compound based on a screening of >1,000 compounds in a lead optimization program. AP30663 has consistently shown the wanted technical quality and necessary properties in various *in vitro* and *in vivo* models, some of which are presented in Bentzen et al., 2020 In the current study we document a pronounced effect on atrial refractoriness by K_Ca_2 inhibition with AP30663. Importantly, we have shown that AP30663 can convert AF to a sinus rhythm in a pig model of AF where existing pharmacotherapy fails. This gives reason to hope that I_KCa_ inhibition with AP30663 might also prove effective in a clinical setting.

K_Ca_2.2 and K_Ca_2.3 subunits are present in both porcine atria and ventricles, but they seem to play a functionally greater role in the atria compared to the ventricles when it comes to repolarization (Diness et al., 2017). In the current study we document a pronounced effect on atrial refractoriness by K_Ca_2 inhibition with AP30663. It would not be unreasonable to expect some degree of QT prolongation based on both the presence of K_Ca_2 mRNA in the ventricles and the fact the AP30663 blocks the hERG channel with an IC_50_ of 4–15 µM (Bentzen et al., 2020), but the clinical development program has so far demonstrated minor impact on QT intervals at expected supratherapeutic doses for atrial efficacy.

### Future Perspectives and Translation to Man

The translation of findings from animal experiments to man will be crucial for AP30663 to become a useful medication. In man, an association between SK channels and AF has been shown in genome-wide association studies concluding that common variants in the genes encoding K_Ca_2.2 and K_Ca_2.3, are associated with AF (Ellinor et al., 2010; Ellinor et al., 2012; Christophersen et al., 2017). Preliminary evidence suggests that I_KCa_ is increased in patients with paroxysmal AF compared to patients in SR, and I_KCa_ inhibition by the peptide apamin was associated with a fourfold increased action potential prolongation in atrial cells from patients in paroxysmal AF (Zhou et al., 2014). I_KCa_ inhibition with NS8593 prolonged refractory periods in human atrial but not ventricular tissue, both from patients in sinus rhythm (SR) and with more than six months of AF (Skibsbye et al., 2014). Increased knowledge about the underlying electro-mechanical remodeling may help understanding of how to select patients who are still responsive to treatment and possibly as to which pharmacological agent and dose could be relevant.

A proof of concept study for AP30663 in patients was begun in 2019 after a successful completion of a phase I study. In parallel, a development program aiming at reformulating AP30663 into an oral formulation is at an early stage, and in addition, the search for another K_Ca_2 inhibitor compound suitable for maintaining sinus rhythm has started.

The properties demonstrated by AP30663 and other SK inhibitors, now used as tool compounds, presently speak in favor of K_Ca_2 inhibition as a novel and interesting concept that is of interest to be tested in man.

A positive proof of concept study in intravenous cardioversion in man would also serve as a proof of concept of the K_Ca_2 inhibition and would be an important trigger for the selection and development of a K_Ca_2 inhibitor also for maintenance of sinus rhythm. At present, a consistent and positive finding in animals has been that reinduction of AF is difficult once conversion to sinus rhythm takes place.

### Limitations

The animals used in the current study were young, whereas patients with AF are usually old. This, and the fact that only female animals were used may limit translatability.

As often is the case in this kind of research, the sample size is a limitation since the study is only powered to detect relatively large and uniform changes in the parameters that are analyzed.

When measuring the AERP after only 10 S1 stimuli the AERP might not have adjusted to the new HR. It is therefore possible that part of the AERP prolongation can be ascribed to a slowing of HR.

The PK data were obtained in anesthetized pigs whereas all the other data were generated in conscious pigs.

### Conclusions

AP30663 has shown properties in animals that would be of clinical interest in man, pending good translation of the findings, and the development program continues. In addition, a positive proof of concept study in intravenous cardioversion in man would also support the K_Ca_2 inhibition concept *per se* and would encourage the development of an oral formulation of AP30663 for rapid oral cardioversion as well as a K_Ca_2 inhibitor with longer half-life suitable for maintenance treatment.

## Data Availability Statement

All datasets generated for this study are included in the article/**Supplementary Material**.

## Ethics Statement

All animal studies were performed under a license from the Danish Ministry of Environment and Food (license No. 2014-15-0201-00390) and in accordance with the Danish guidelines for animal experiments according to the European Commission Directive 86/609/EEC.

## Author Contributions

JK, TS, and LA contributed with acquisition, interpretation, and analysis of data. US, MG, and NE contributed with the conception and design of the study. BB and JD wrote the first draft of the manuscript and interpreted and analyzed the data and contributed with the conception and design of the study. JD also contributed with acquisition, interpretation, and analysis of data. All authors contributed to manuscript revision, read, and approved the submitted version.

## Funding

This work was supported by the Innovation Fund Denmark, Wellcome Trust [Grant Number 100406/Z/12/Z].

## Conflict of Interest

BB, JK, TS, NE, US, LA, MG, and JG are employed by and/or have interests in Acesion Pharma and/or are inventors of Acesion Pharma patents within the field of SK channels.

The remaining author declares that the research was conducted in the absence of any commercial or financial relationships that could be construed as a potential conflict of interest.
