# Supplementary material for: The KCa2 Channel Inhibitor AP30663 Selectively Increases Atrial Refractoriness, Converts Vernakalant-Resistant Atrial Fibrillation and Prevents Its Reinduction in Conscious Pigs
Source: Front Pharmacol. 2020 Feb 28;11:159. doi: 10.3389/fphar.2020.00159 (PMC7059611; doi:10.3389/fphar.2020.00159)
Supplement: Supplementary file 1 [file DataSheet_1.docx]

Supplementary Material

# Supplementary Data

Supplemental methods, materials and results to:

Diness et al. **The KCa2 channel inhibitor AP30663 selectively increases atrial refractoriness, converts vernakalant-resistant atrial fibrillation and prevents its reinduction in conscious pigs**

[Supplementary materials 1](#_Toc24355544)

[Table 2: Equipment 1](#_Toc24355545)

[Table 3: Drugs 5](#_Toc24355546)

[Table 4: Software 8](#_Toc24355547)

[Supplementary methods 9](#_Toc24355548)

[Induction of AF, cardioversion of AF and reinduction of AF in pigs 9](#_Toc24355549)

[Supplementary results 10](#_Toc24355550)

[Table 5: AF conversion 10](#_Toc24355551)

# Supplementary materials

## Table 2: Equipment

| **Equipment** | **Producer** | **Model** | **Rationale for choosing this** |
| --- | --- | --- | --- |
| AERP pacemaker | Biotronik, Berlin, Germany | Etrinsa, DR-T | Implantable pacemaker capable of wireless programming and manual recordings of AERP. |
| AF Neurostimulator | Medtronic, Dublin, Ireland | Synergy versitrel | Implantable stimulator capable of delivering sufficiently fast and long electrical impulses for cardiac tachypacing. |
| AF Neurostimulator | Medtronic, Dublin, Ireland | Itrel 3 | Implantable stimulator capable of delivering sufficiently fast and long electrical impulses for cardiac tachypacing. Slightly smaller than the synergy versitrel making it easier to implant. No longer in production. |
| Sterile filter | Thermo Scientific, Waltham, Massachusetts, USA | Nalgene, Rapid flow 90 mm filter unit, 250-500 ml. | State of the art product for sterile filtration. |
| Implantable leads | St Jude Medical, Little Canada, Minnesota, US | Tendril (2088T/58 cm) | State of the art electrical lead with active fixation. Used routinely for patients. |
| Biotronik pacemaker programmer |  |  |  |
| Medtronic neurostimulator programmer |  |  |  |
| Televet, Holter monitor | Engel Engineering Service GmbH, Heusenstamm, Germany | Televet-100 | State of the art Telemetric ECG System for veterinary medicine |
| Infusion pump | Shenzhen Shenke Medical Instrument Technical Dev  elopment Co.,Ltd., Shenzhen P.R.China | SK-500I syringe pump | Syringe pump capable of delivering up to 500 ml/h |
| 50 ml syringe | Medical Surgical Systems BD, Albertslund, Denmark | BD Plastipak 50 ml Luer-Lok syringe | Standard 50 ml Luer-Lok syringe compatible with most syringe pumps. |
| Patient Monitor | Agilent, Glostrup, Denmark, | Viridia | Monitoring of ECG, Blood pressure SP0_2_ and temperature of the pig during surgery. |
| Respirator | Demeca, Rødovre, Denmark | Siesta i TS | Respiration of the pig during surgery. 500 mL/min 0_2_ and 2500 mL/min air. |
| Infusion pump | B. Braun Melsungen AG,  Melsungen, Germany | Infusomat space | Pump for infusion of Propofol. |
| Infusion pump | B. Braun Melsungen AG, Melsungen, Germany | Perfuser compact | Syringe pump for infusion of fentanyl. |
| Infusion pump | Heska,  Loveland, Colorado, USA | Vet/IV | Infusion pump for saline solution. |

## Table 3: Drugs

| **Drug** | **Producer** | **Strength** | **Dosing** | **Rationale** |
| --- | --- | --- | --- | --- |
| **Test drug** | | | | |
| AP30663 | Acesion Pharma, Copenhagen, Denmark/ Syngene, Bangalore, India | Batch numbers:  0002, 0003, 0004, 0005, 1406, 1407  5 mg/ml solution in vehicle I  10 mg/ml solution in vehicle II | 5-25 mg/kg | See introduction |
| **Other drugs** | | | | |
| Zoletil pig mix | Unit for experimental medicine, Copenhagen University | Solution for injection, 250 mg dry tiletamin+zolazepam, 6.5 ml xylazine 20 mg/ml, 1.25 ml ketamine 100 mg/ml, 2.5 ml butorphanol 10 mg/ml, and 2 ml methadone 10 mg/ml | 0.1 ml/kg, IM | Used for initial sedation and anaesthesia of the pigs. |
| Gentacoll® patches | Swedish Orphan Biovitrum | 32.5 mg gentamycin in 5x5 cm patch | 32.5 mg in implantation pocket. | Broad-spectrum antibiotic to avoid infection in the implantation pocket. |
| Propolipid | Fresenius Kabi | Liquid for infusion, emulsion of 10 mg/ml propofol. | 15 mg/kg/h, IV | Anaesthetic agent |
| Fentanyl | “B. Braun”,  “2Care4”, or “Sandoz” | Solution for injection, 50 µg/ml | 0.5 µg/kg/h | Opioid. Anaesthetic and analgesic agent. |
| Norostrep Vet. | ScanVet Animal Health A/S, Fredensborg | Liquid for infusion, emulsion of 200.000 IU (200 mg)/ml. | 2 ml on the pacemaker just before implantation. | Antibiotic for penicillin- and dihydrostreptomycin-vulnerable bacteria. |
| Curamox prolongatum Vet. | Boehringer Ingelheim | Liquid for infusion, emulsion of 150mg amoxicillin/ml | 15 mg/kg, IM | Broad-spectrum antibiotic for amoxicillin-vulnerable bacteria with prolonged effect (>48 h) |
| Metacam | Boehringer Ingelheim | 15 mg meloxicam/ml | Oral suspension  0.5 mg/kg PO daily for three days after implantation | NSAID for post operational pain. |
| Clamoxyl Vet. | Orion Pharma Animal Health A/S | 510 mg amoxicillin/g | Powder for oral solution 40 mg/kg PO daily for 5 days after implantation | Broad-spectrum antibiotic for amoxicillin-vulnerable bacteria. |

All other drugs than the test item used in this experiment are commercially available and sourced from the open market within the European Union. The zoletil pig mix is not a marketed combination, and was prepared by the Unit for Experimental Medicine at Copenhagen University.

## Table 4: Software

| **Software** | **Supplier** | **Rationale** |
| --- | --- | --- |
| GraphPad Prism 8.2.0 | GraphPad Software, Inc. | Software for simple statistical analyses and for graphical representations of data. |
| LabChart 7.3.7. | ADInstruments | A platform for multiple recording devices to work together, allowing the acquisition of biological signals from multiple sources simultaneously. Allows for semi-automated analyses of ECGs. |
| Televet ECG software 6.0.0 | Engel Engineering Services GmbH, Germany | Works with the Televet100 Holter monitor for acquisition of ECGs from conscious pigs. |

# Supplementary methods

## Induction of AF, cardioversion of AF and reinduction of AF in pigs

After at least 7 days recovery, the pacemakers were turned on and the RA was paced 420 beats per minute (bpm). The pigs were medicated with digoxin (250 µg/day) throughout the study starting 2-4 days before turning on the pacemaker in order to prevent heart failure symptoms following pacing. After 7 days of pacing, the ECG was monitored while the pacemaker was turned off. If a pig had AF lasting for at least 10 minutes it was assigned to vernakalant treatment. Vernakalant (4 mg/kg) was infused over 10 minutes and the pig was monitored for an additional 30 minutes. After this, or if AF converted at any point, the pacemaker was turned on (420 BPM) again. If AF was converted at any point, the pig was paced 420 bpm for 1-7 more days and the procedure was repeated. When AF could no longer be converted to sinus rhythm by vernakalant, the pig was paced 420 BPM for another 24-48 hours to allow vernakalant to be cleared from the system (half-life 2-3 hours). Following this, the pacemaker was turned off under ECG monitoring. When AF lasting for at least 10 minutes had been confirmed, treatment with AP30663 was commenced.

The compound was infused with a rate of 20 mg/kg/h over 60 minutes.

If AF converted within this period, burst pacing with 50 Hz was applied thrice with 2 times rheobase current intensity. Cardioversion was considered successful if sustained AF was converted. Protection against reinduction of AF was considered successful if no episodes of AF lasting for more than 10 minutes could be re-induced by burst pacing. If any re-induced AF episode lasted more than 10 minutes and cardioverted at a later stage, burst pacing was applied again and if no episodes of AF lasting for more than 10 minutes could be re-induced by burst pacing the pig was considered protected against reinduction of AF.

# Supplementary results

## Table 5: AF conversion

| **Pig ID** | **Days of A-TP until first detection of persistent AF** | **Days of A-TP until vernakalant-resistant AF** | **Days of A-TP until AP30663** | **Cardioversion dose and corresponding calculated free plasma concentration** | **Time to cardioversion** | **Protection dose** | **Tolerability** |
| --- | --- | --- | --- | --- | --- | --- | --- |
| 429 (2015) | 18 | 18 | 20 | 5.25 mg/kg  1.23 µM | 18 min | 5.5 mg/kg | Well tolerated |
| 135 (2016) | 21 | 21 | 24 | None | None | None | Well tolerated |
| 275 (2016) | 9 | 9 | 10 | None | NA | None | Well tolerated |
| 281 (2016) | 12 | 12 | 16 | 14.5 mg/kg  1.36 µM | 43.5 min | 14.5 mg/kg | Well tolerated |
| 304 (2016) | 13 | 13 | 15 | None | NA | NA | Well tolerated |
| 303 (2016) | 21 | 21 | 24 | 6.3 mg/kg  1.25 µM | 19 min | 7 mg/kg | Well tolerated |
| 382 (2016) | 21 | 21 | 22 | 5 mg/kg  1.17 µM | 15 min | None | Well tolerated |
| 381 (2016) | 15 | 15 | 17 | 6.7 mg/kg  1.26 µM | 20 min | 11.7 mg/kg | Well tolerated |
| 380 (2016) | 15 | 15 | 21 | 20 mg/kg  1.36 µM | 60 min | None | Well tolerated |
| 406 (2016) | 18 | 22 | 23 | None | NA | NA | Well tolerated |
